# New Magnetostrictive Transducer Designs for Emerging Application Areas of NDE

**DOI:** 10.3390/ma11050755

**Published:** 2018-05-08

**Authors:** Sergey Vinogradov, Adam Cobb, Jay Fisher

**Affiliations:** Southwest Research Institute, 6220 Culebra Rd, San Antonio, TX 78238, USA; adam.cobb@swri.org (A.C.); jay.fisher@swri.org (J.F.)

**Keywords:** magnetostrictive transducers, guided waves testing, reversed Wiedemann effect, nonlinear harmonics

## Abstract

Magnetostrictive transduction has been widely utilized in nondestructive evaluation (NDE) applications, specifically for the generation and reception of guided waves for the long-range inspection of components such as pipes, vessels, and small tubes. Transverse-motion guided wave modes (e.g., torsional vibrations in pipes) are the most common choice for long-range inspection applications, because the wave motion is in the plane of the structure surface, and therefore does not couple well to the surrounding material. Magnetostrictive-based sensors for these wave modes using the Wiedemann effect have been available for several years. An alternative configuration of a sensor for generating and receiving these transverse-motion guided waves swaps the biasing and time-varying magnetic field directions. This alternative design is a reversed Wiedemann effect magnetostrictive transducer. These transducers exhibit a number of unique features compared with the more conventional Wiedemann sensor, including: (1) the use of smaller rare earth permanent magnets to achieve large, uniform, and self-sustained bias field strengths; (2) the use of more efficient electric coil arrangements to induce a stronger time-varying magnetic field for a given coil impedance; (3) beneficial non-linear operating characteristics, given the efficiency improvements in both magnetic fields; and (4) the ability to generate unidirectional guided waves when the field arrangement is combined with a magnetically soft ferromagnetic strip (patch). Reversed Wiedemann effect magnetostrictive transducers will be presented that are suitable for different inspection applications, one using electromagnetic generation and reception directly in a ferromagnetic material, and another design that integrates a magnetostrictive patch to improve its efficiency and enable special operating characteristics.

## 1. Introduction

The use of guided waves for the long-range inspection of components is a rapidly growing segment of the nondestructive evaluation (NDE) service business [1,2,3]. Magnetostrictive sensors have been shown to be very effective transduction tools for the guided wave testing of pipes and plates [4,5]. However, there is still a great demand for enhanced sensor characteristics or specific requirements for sensor design compared to what is possible with most conventional guided wave sensor offerings. For example, some applications require especially low profile or small sensors [6]. Another example is the need for sensors for the structural health monitoring (SHM) of components operating at elevated and extreme temperatures. A very robust sensor design is needed that is capable of surviving the high temperatures as well as the stresses induced by thermal cycling [7,8,9]. A third example is the guided wave testing of components with high attenuation, such as buried or coated pipes, for which higher transmitted signal amplitudes would be very beneficial [10,11]. There is also interest in utilizing higher frequency guided waves for estimations of flaw depth using multi-mode testing [12,13] or multi-frequency guided waves for the characterization of notch/crack type of anomalies. These types of anomalies demonstrate pronounced variations in signal amplitude as a function of the used frequency range [14,15].

A comprehensive review of magnetostrictive patch transducers (MPTs) and different methods of magnetostrictive transduction was given by Kim and Kwon [16]. In this paper, the authors used the abbreviation MsT (magnetostrictive transducer) for magnetostrictive sensors that are used for the generation of both longitudinal and torsional vibrations using Joule and Wiedemann effects and their inverse effects, sometimes known as the Villari and Mateucci effects, for receiving the signals.

This paper will describe a novel transducer that was invented in 2008 [17]. The authors consider the principle of transducer operation to be classified specifically as a reversed Wiedemann effect. The transducer was given the abbreviation MsT in an earlier publication on this topic by Vinogradov in [18]. This is why in this paper, the abbreviation ‘MsT’ will be explicitly used for transducers utilizing the reversed Wiedemann effect that are used for the generation of transversal vibrations (torsional in a cylindrical structure and shear horizontal in plate structures).

Reversed Wiedemann effect transducers were found to address the high challenge areas described earlier very well. Note that not all of the characteristics of magnetostrictive transducers of the MsT-type have been completely understood in terms of numerical models, but some experimental data representing the transducer performance were presented by Vinogradov, Cobb and Light [19]. This paper discusses the results that were obtained earlier, and also the results of additional research that has been conducted on the same topic.

## 2. Wiedemann Effect

**Figure 1:**
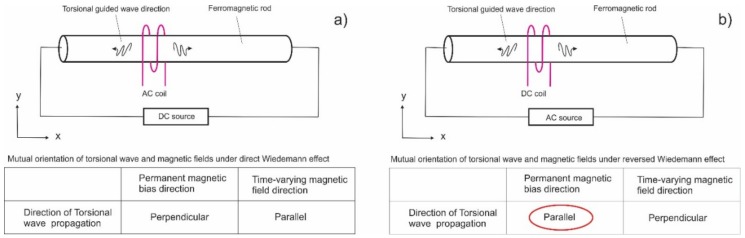
Two alternative implementations of Wiedemann effect: (**a**) direct Wiedemann effect when the circumferential field is permanent, and (**b**) a reversed Wiedemann effect when the circumferential field is time-varying.

The Wiedemann effect was discovered more than a century ago by Gustav Wiedemann [20]. The general definition is the twisting of a ferromagnetic rod when an electric current is passed down its length (creating a circumferential magnetic field) while the rod is simultaneously placed in a longitudinal magnetic field. One common use of this effect is the generation of torsional guided wave modes by making one of these fields in the rod structures time-varying. Figure 1 shows two alternative implementations of the Wiedemann effect: (a) a permanent circumferential field (propagation perpendicular to this bias)—the conventional Wiedemann effect, and (b) a time-varying circumferential field (propagation parallel to the axial bias)—a reversed Wiedemann effect.

Magnetostrictive sensors have been designed that generate waves either in highly-magnetostrictive ferromagnetic strips (which are mechanically coupled to the part under test) or directly in the part under test. These sensors can use either the direct or the reversed Wiedemann effect. The direct Wiedemann effect is a more common implementation.

It should be noted that the magnetically-induced forces exist not only in rods but also in hollow cylindrical shells. Further development of magnetostrictive electromagnetic acoustic transducers (EMATs) was conducted by Thompson [21], with the majority of the probes utilizing the propagation of generated transverse vibrations perpendicular to this permanent magnetic bias. MsTs belong to the group of magnetostrictive EMATs utilizing the less common configuration, with the transverse vibrations propagating parallel to the magnetic bias direction.

## 3. Invariant Properties of Shear Wave Magnetostrictive EMATS

Practical applications of shear wave EMATS require transversal vibrations to propagate along one selected axis. For example, for the long-range guided wave testing of pipes, the desired propagation directions will be parallel to the pipe axis (in both positive and negative directions), and unwanted directions will be circumferential. Some applications, such as SHM, might utilize propagation in orthogonal directions to monitor a larger area from one probe position. Inherently, direct and reversed Wiedemann effect EMATs have exactly the same principal coil arrangement: one coil provides a time-varying magnetic field that is perpendicular to another coil/magnet that provides a permanent magnetic bias, with both coils generating in-plane magnetic fields. In this sense, the only difference between a direct and a reversed Wiedemann effect is how the coils are oriented in reference to the desired wave propagation direction. If the directivity of the EMATs is not a strict requirement, shear wave magnetostrictive EMATSs could exhibit an invariant property in reference to the wave propagation direction so long as the following condition is satisfied: applying permanent and time-varying magnetic fields results in an equivalent magnetic domain re-alignment in two mutually perpendicular directions. Satisfying this condition is easier when the material is magnetically soft and structurally and magnetically isotropic. This paper is focused on reversed Wiedermann effect sensors. Some practical designs of direct Wiedemann effect sensors will also be discussed.

## 4. Sensors Utilizing Direct Wiedemann Effect

**Figure 2:**
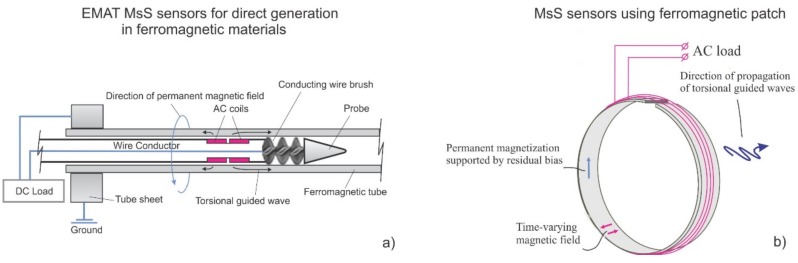
Sensors utilizing a direct Wiedemann Effect: (**a**) electromagnetic acoustic transducer (EMAT) heat exchanger probe for the guided wave testing of carbon steel heat exchanger tubing, (**b**) magnetostrictive sensor (MsS) used for the guided wave testing of pipes.

Some common implementations of direct Wiedemann effect sensors for industrial structures are magnetostrictive sensors (MsS) for pipeline inspection and EMAT implementations for ferromagnetic tube inspection. Figure 2 shows (a) the design of an EMAT probe for heat exchanger tubing and (b) the design of an MsS probe that is used for guided wave testing of pipes. The EMAT configuration for tubing utilizes a biasing magnetic field created by passing a direct current (of the order of 15 Amperes) along the length of the tube, and a time-dependent axial magnetic field produced by passing a pulsed current through a coil inside or outside the tube. Since the magnetostrictive properties of carbon steel tubes can vary significantly, even at different locations in the same tube, the signal quality of this arrangement might vary. Based on field experience, the presence of oxides on the tube surface typically improves the probe performance. When the ferromagnetic properties are favorable, good signal quality has been reported in a wide frequency range (32–128 kHz) on carbon steel heat exchanger tubes [22]. It should be noted that these probes cannot be used in magnetically soft materials such as Duplex^®^ or Seacure^®^ because the RF coils introduce a residual magnetic field along the length of a tube. This residual field interacts with the dynamic magnetic field created by the same coil to create unwanted longitudinal mode guided waves. Due to this effect, reversed-Weidemann style sensors are used in these alloys, as discussed in a later section.

MsS sensors for pipeline testing can conceptually employ the same configuration as an EMAT (circumferential bias field and axial time-varying field). The main difference is the addition of a magnetostrictive material that is attached to the pipeline, accentuates the magnetostrictive effect, and also allows operation on non-ferromagnetic materials. Typically, the circumferential field is created by moving a magnet along the strip material to induce a residual magnetic field.

Although sensors that have been designed as described above have been used for many years for tube and pipeline inspection, there are some challenges that exist with these sensors:

- For larger sensors, the winding of the RF coils for the time-varying field becomes prohibitively long, and the electrical impedance becomes very high; this can be challenging for existing electronics, because the dynamic field strength is proportional to the current in the RF coil at a fixed frequency.
- It can be difficult to introduce a strong and/or uniform magnetic bias for larger sensors, given the size of the area to magnetize.
- Most commercial MsS sensors use residual magnetic biasing that is vulnerable to external magnetizing fields and stresses applied to the strip.

Some of these problems have been addressed with more advanced designs. For example, the problem with maintaining the magnetic bias was resolved in the MsS sensor configurations by including a DC winding to act as an electromagnet [23,24]. Not only does this allow larger signal amplitudes, these sensors were also shown to stay functional at temperatures close to the Curie point, 930 °C [9]. 

## 5. Reversed Wiedemann Effect Sensors

**Figure 3:**
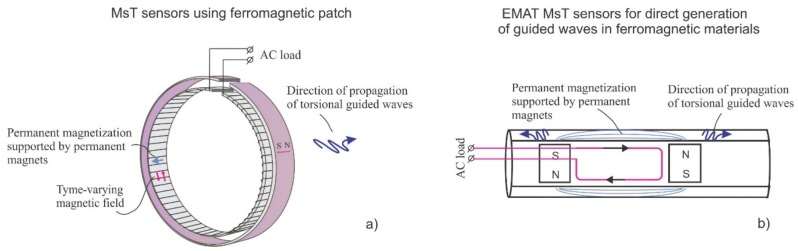
Sensors utilizing a reversed Wiedemann effect: (**a**) a magnetostrictive transducer (MsT) for the guided wave testing of pipes; (**b**) an EMAT MsT for the testing of heat exchanger tubes.

MsTs were developed as an alternative to direct Wiedemann effect sensor options. The physics of operation are unchanged. However, the magnetic and structural anisotropy of the ferromagnetic material should be considered, because it will influence the transducer performance. Figure 3 shows two different reversed Wiedemann effect sensor designs: (a) a MsT utilizing a ferromagnetic strip that is used for testing pipes, and (b) an EMAT MsT that is used for testing tubes.

MsTs for piping, as illustrated in Figure 3a, have several notable engineering advantages over MsSs. Since guided wave sensors are typically much longer in the particle motion direction than transverse to the particle motion direction, the reversed Wiedemann effect style means that the magnetic biasing length is relatively short. Rare earth magnets are more readily available in this size to induce stronger and more consistent bias fields. Also, efficient solenoidal coil designs can be used to reduce impedance, which allows the use of simpler electronics, reducing power requirements or increasing signal strength.

The EMAT MsT for heat exchanger tube inspection that is shown in Figure 3b has been found to be advantageous for the guided wave testing of magnetically soft ferromagnetic alloys such as SeaCure and Duplex. This is because the RF coil does not create any time-varying magnetic field in the axial direction of the tube (only a circumferential magnetic field is generated). The results of successful field trials on 5/8 inch OD SeaCure tubing using the fundamental T(0.1) mode have been previously discussed [25,26].

Another interesting version of an omnidirectional MPT probe was proposed by Seung, Kim and Kim [27]. We believe that this probe falls into a category of reversed Wiedemann effect transducers, because its permanent magnetic bias direction is parallel to the shear horizontal wave propagation direction.

## 6. MsTs with Special Functionality: Generation of Higher Order Harmonics

An MsT functions based on the interaction of two magnetic fields—one dynamic and the other static—within the magnetostrictive strip. The dynamic magnetic field is typically much weaker than the static field; the static bias field strength is designed to provide the best performance in generating guided waves. However, it is possible to adjust the relative strength of the two magnetic fields to produce interesting and potentially useful operational characteristics. For example, one possible configuration change results in the capability to produce higher order harmonics for the input frequency signal. One scenario where this would be advantageous is when the transmitter circuitry does not have sufficient bandwidth to generate higher frequency signals for a given inspection application.

To explain how this modification works, recall that a magnetic field applied to an unstressed/unbiased magnetostrictive material will strain in one direction regardless of the polarity of the applied magnetic field. In other words, reversing the magnetic field polarity will produce the same strain vector in the material. The effect of this is that applying a time-varying magnetic field in this unbiased configuration causes time-varying strains, but the frequency of the strains are twice that of the input magnetic field. One essential purpose of the static field bias in an MsT is to induce a baseline strain in the strip so that the dynamic field produces both positive and negative strains relative to the baseline strain state. Thus, the frequency of the guided waves that are generated using an MsT is the same as the input signal to the sensor.

**Figure 4:**
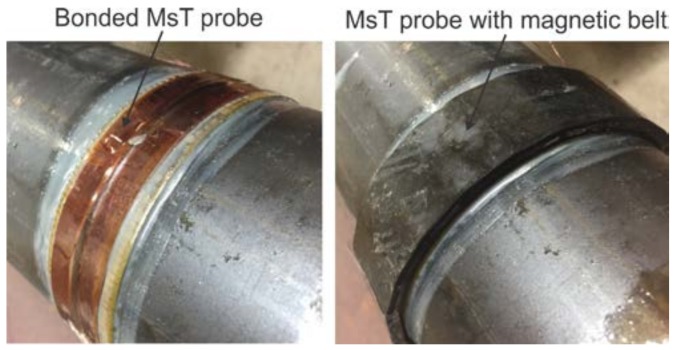
Photograph of pipe and MsT probe used for harmonic generation experiments.

**Figure 5:**
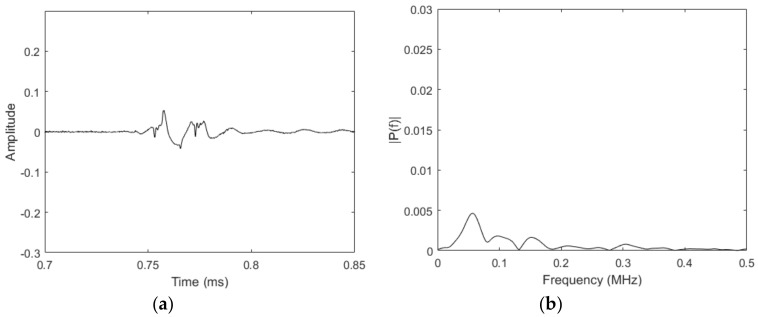
Acquired signal (**a**) together with its FFT (**b**) for a 50-kHz excitation with a bias magnet liftoff of 4 mm.

Based on this, it should be possible to reduce the magnetic bias field strength and produce second order harmonics. Testing has been conducted to confirm this result using an MsT transducer. For this experiment, an 89 mm outer diameter, 6.35-mm thick wall (T(0,1) cutoff > 350 kHz), 3.65-m long pipe was used, as shown in Figure 4. A 19-mm wide magnetostrictive strip with the dynamic field coil wrapped around the strip with a 3-mm pitch was bonded to the pipe (2.1 m from the far end). The strip was biased with a magnetic belt arranged to provide an axial field and placed on top of the strip with 4-mm liftoff; the amount of liftoff between the biasing magnets and the magnetostrictive strip significantly reduced the bias field strength compared to the normal static field configuration. The magnetic field introduced into the strip was measured to be approximately 3 kGauss, whereas a typical bias field strength is 7 kGauss. The experimental process was to examine the frequency content of a reflection from the far end of the pipe from a 50-kHz torsional guided wave. The acquired signal from the reflection from the end of the pipe and its fast Fourier transform (FFT) are shown in Figure 5.

**Figure 6:**
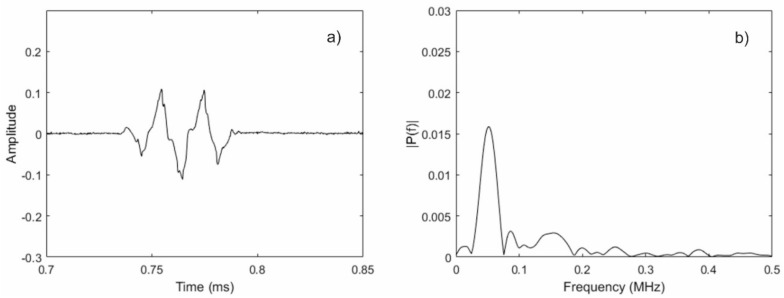
Acquired signal (**a**) and its FFT (**b**) for a 50-kHz excitation frequency with no bias magnet liftoff.

From Figure 5, a peak at the input center frequency of 50 kHz can be observed, along with an additional peak near 100 kHz. In other words, the second harmonic was generated using only a single frequency input signal. Unfortunately, the signal-to-noise ratio (SNR) of the data was very poor; for comparison, Figure 6 shows data collected using the same sensor setup where the liftoff between the magnets and the magnetostrictive strip was removed, but all of the other settings remained the same. The reason for the reduced performance of the MsT at the low bias field strength configuration is that the magnitude of the magnetostrictively-induced strain was not purely dependent on the magnitude of the dynamic field. In reality, the second major purpose of the static bias was to improve the amplitude of the generated guided wave signals. It can be shown that the performance of similar EMATs is improved by biasing the sensor to an operating point where the slope of the magnetostrictive strain versus the applied field curve is high. This is intuitive because, by biasing the sensor in this fashion, it allows for the largest possible strain change for a given dynamic field strength. Thus, reducing the static bias field strength to produce second harmonic waves is not a viable strategy, given the simultaneous reduction in sensor performance.

It is important to maintain a conventional bias field strength so that the sensor can realistically act as a sensor for inspection. The only other change that is possible is to adjust the dynamic field in such a way to produce higher frequency components. For similar EMATs, the dynamic field strength is not large enough to cause the maximum possible strain. In other words, the dynamic field causes positive and negative strains in the material without hitting any theoretical strain limits. However, it stands to reason that if the dynamic field is large enough, it should be possible to achieve the maximum and minimum theoretical strains possible in the material.

**Figure 7:**
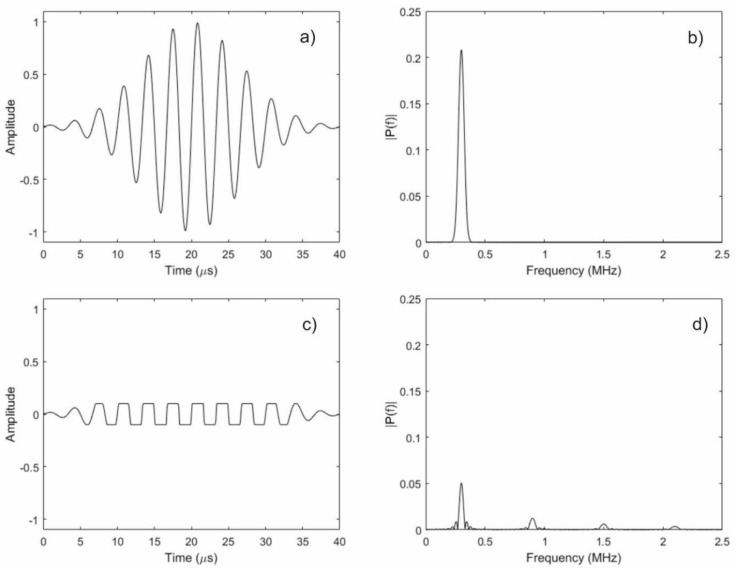
Illustration of the effect of clipping a sinusoid on its frequency content. The top two figures show a 300-kHz sinusoid with a Gaussian envelope applied (**a**), and its associated spectrum (**b**). The bottom two figures show the same sinusoid clipped to be within the range of −0.1 to 0.1 (**c**), and its associated spectrum (**d**). Note how the unclipped spectrum has its energy concentrated near 300 kHz, whereas the clipped spectrum has energy at the odd harmonics of 300 kHz (i.e., 300 kHz, 900 kHz, 1.5 MHz, etc.).

It is clear that the largest signal amplitudes should occur when the dynamic field is large enough to produce the maximum possible strain. However, if the dynamic field strength is increased beyond this level, there will be moments during transmission where the strain under the sensor achieves the maximum strain state, and remains there until the dynamic field level is reduced. More simply, the time-varying strain caused by the sensor is a clipped sinusoid instead of a pure sinusoid. The significance of this difference is that the Fourier transform of a clipped sinusoid has a dominant frequency component at the same frequency as the unclipped sinusoid, as well as every odd harmonic. Figure 7 illustrates the effect of clipping a sinusoid on its frequency content. Thus, it should be possible to produce higher frequency waves using a sensor that is similar to the ones described in this paper if the dynamic field strength is increased.

**Figure 8:**
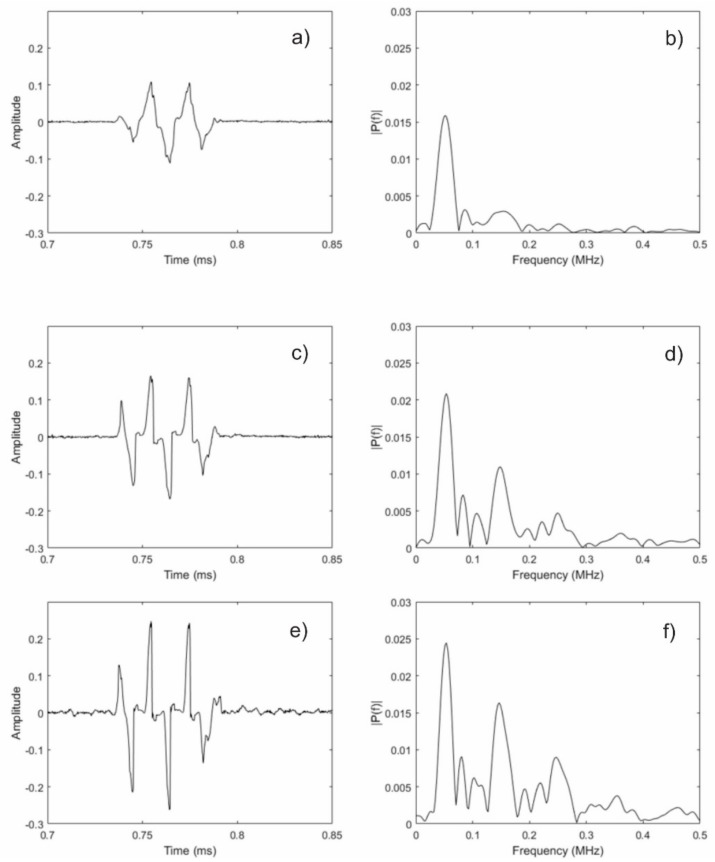
Acquired guided wave reflection signals from the end of the pipe with no magnet liftoff and at different transmitter current settings: (**a**,**b**) show the normal current level signal and resulting FFT, respectively; (**c**,**d**) show the doubled current signal and FFT, respectively; (**e**,**f**) show quadrupled current signal and FFT, respectively.

For all practical purposes, it is not realistic to use high enough currents to achieve the required dynamic field strengths with a conventional EMAT. However, an MsT uses a solenoidal winding that encircles the magnetostrictive strip. This improves the efficiency of generating the dynamic field for a given transmit signal current. To test this concept, an experiment was performed using the same sensor that is shown in Figure 4. The biasing magnets were installed without liftoff to achieve the correct biasing state. The transmit signal current was then increased beyond that which is typically used with an MsT. Figure 8 shows the results of this experiment. At a normal transmit current (Figure 8a,b), the received signal looks slightly triangular, and the frequency content is almost exclusively near 50 kHz, which is the transmit signal frequency. Note that the first result shown is the same data that is shown in Figure 6, but replotted here to aid in visual comparison. The second and third results shown are with the transmit signal current doubled (Figure 8c,d) and quadrupled (Figure 8e,f), respectively. As can be seen, the received signal shaped is distorted at these increased currents. This distortion is a result of the clipped structure of the guided wave; the receive mechanism for this sensor type is only sensitive to the changing strain states, not to the static strain conditions. Examination of the frequency content reveals the odd multiple harmonic peaks at 150 kHz, 250 kHz, and 350 kHz. It also should be noted that since there is a sufficient bias field, the second harmonic that was seen before is not present, as expected.

These capabilities to generate harmonics of the applied signal could be beneficial in certain situations, e.g., where the electronics cannot generate the desired frequency, or where multiple frequencies are desired. Examples of the utilization of a non-linear ultrasound for practical NDE include the detection and characterization of cracks based on closed–open criteria [28] and the microstructural damage of materials [29]. Examples of the utilization of MsTs and non-linear harmonics for the discrimination of through-wall anomalies in nuclear fuel rods were discussed by Fisher et al. in [6] and by Vinogradov in [30].

## 7. MsTs with Special Functionality: Unidirectional Operation

**Figure 9:**
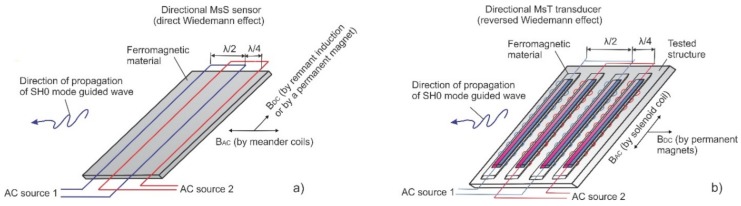
Directional transmission of guided waves: (**a**) utilizing two quarter-wavelength spaced meander coils (each coil has two conductors that are separated by one half of the wavelength); (**b**) utilizing two quarter-wavelength spaced MsT type transducers (each transducer in turn has two channels separated by one half of the wavelength and connected to reject the unwanted direction).

The second design discussed here is a single winding unidirectional MsT transducer. This type of transducer is desirable because direction control currently requires the use of at least two transducer positions or two transducers with an active phasing of the excitation signal and/or signal processing after data collection. When using two transducers with active phasing, a typical amplitude ratio between signals traveling in the wanted and unwanted direction is of the order of 12–15 dB. Each of the two transducers is typically further split into multiple segments that are spaced a half wavelength apart. This is similar to a meander coil EMAT; each meander repetition reinforces the generation of guided waves at a specific wavelength and increases the rejection of other wavelengths. Combining two transducers—each with multiple segments—further improves the direction control capability and the amplitude ratios between the signals traveling in the wanted and unwanted directions; with this arrangement, the amplitude ratio can reach 25–30 dB. Figure 9a shows a direct Wiedemann effect probe with two quarter-wavelength spaced meander coils (each coil has two channels separated by one-half of the wavelength). Figure 9b shows an MsT probe (reversed Wiedemann effect) with two-quarter wavelength spaced transducers (each transducer has two segments that are also separated by half of the wavelength to reject unwanted directions).

The major disadvantage of these probes is that the best direction control performance is achieved only at the correct wavelength. Switching the frequency range (and thus wavelength) requires changing the spacing between segments; this can be difficult when an access to a probe is limited. In addition, active phasing requires multi-channel equipment for operation, which increases power consumption and electronics complexity. Thus, there is a motivation to develop a unidirectional MsT for special applications where broader frequency operation and/or simpler electronics are required.

**Figure 10:**
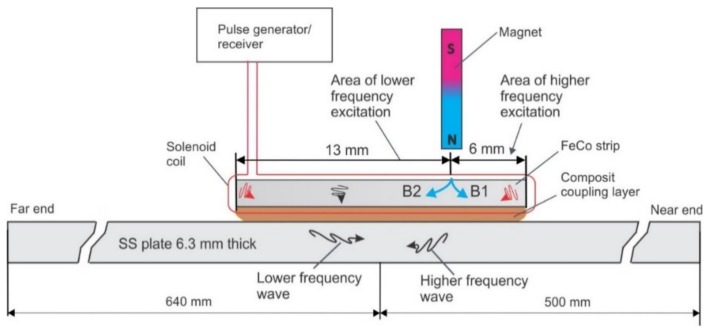
Unidirectional MsT probe arrangement—the ferromagnetic strip is magnetized by a permanent magnet offset from the strip center.

The basic concept explored here for the development of a unidirectional MsT was to offset the permanent magnet to one side of the magnetostrictive strip. Normally, the biasing magnet on the strip uniformly biases the strip; in contrast, the offset divides the magnetostrictive strip into two regions with opposite bias polarization. The expectation is that the strains produced in one region will be in the opposite direction of the other region because of the static field reversal. A conceptual illustration of the setup is shown in Figure 10. This illustration uses a 19-mm wide magnetostrictive strip; the magnet placement divides the strip into two areas, one that is 6 mm wide, and another that is 13 mm wide. While not completely understood at this point, the intent is for the waves that are generated in each section to interfere with one another to control the wave propagation direction.

**Figure 11:**
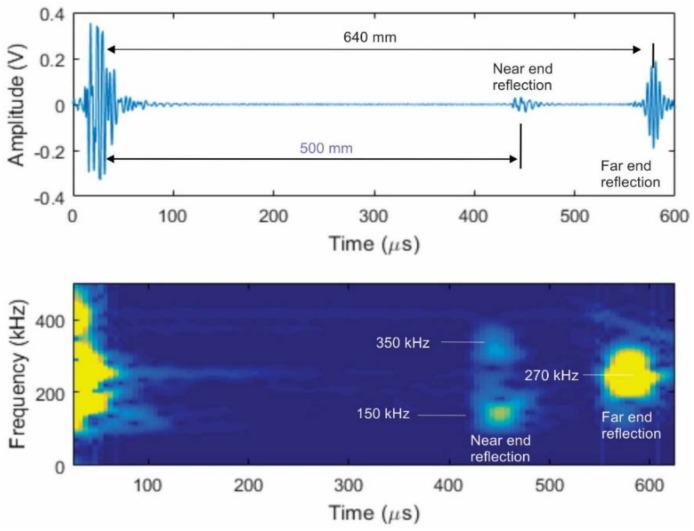
Reflections obtained from the ends of the stainless steel plate with the arrangement shown in Figure 10 (upper image), and the spectrogram of signals obtained from the opposite plate edges (lower image).

A sensor was built following this conceptual drawing and coupled to a 6.3-mm thick stainless steel plate using a shear wave couplant, as shown in the Figure 10. A broadband pulse centered at 300 kHz was applied to the solenoid coil. This sensor was used for both the generation and reception of the guided waves. At this input frequency, shear-horizontal guided waves will be generated. The probe produced waves traveling in both directions, but their frequency content and amplitudes were different, as shown in Figure 11. The current theory is that the waves generated from each active region will have different characteristics in terms of frequency and amplitude. This is because a strip that is much larger than one-quarter of the wavelength that is being generated would self-cancel during generation. Thus, the wider active region should produce a larger signal amplitude while having a lower operating frequency range than in the narrower active region.

**Figure 12:**
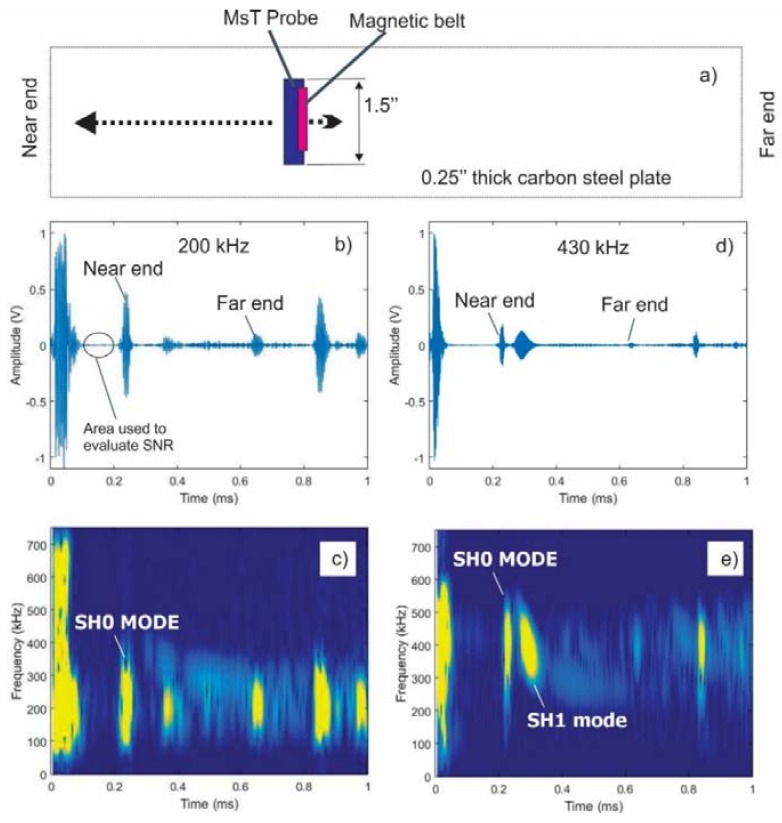
Demonstration of unidirectional probe operation at 200-kHz and 430-kHz center frequencies on a 0.25-inch thick carbon steel plate: (**a**) test arrangement using 1.5-inch wide MsT transducer, (**b**) 200-kHz data, (**d**) 430-kHz data. The spectrograms of signals in panels (**b**,**d**) are shown in (**c**,**e**).

This test confirmed the unidirectional operation of this sensor concept. The higher (>300 kHz) and lower (<150 kHz) frequency information propagated toward the nearest plate boundary, while the central frequency information around 270 kHz propagated predominately toward the far plate boundary. Furthermore, after applying band-pass filtering to the waveform shown in Figure 11, it was determined that the amplitude ratio between the higher frequency signal traveling in the wanted (higher frequency signal) and unwanted (lower frequency signal) directions was near 25 dB. Another test conducted on a 0.25-inch thick carbon steel plate produced an amplitude ratio of the order of 16 dB in the frequency range of 200–430 kHz without adjustment of the magnet position. Figure 12a shows the test arrangement and test results using a 1.5-inch wide MsT transducer. The magnet placement divides the strip into two areas, one that is 6 mm wide, and another that is 18 mm wide. The narrow magnetized region was closest to the near edge of the plate as with the prior example, so the expectation is that the higher frequency components will propagate primarily toward the near edge. Data at a 200 kHz center frequency are shown in Figure 12b, and 430-kHz data are shown in Figure 12d. The spectrograms of the received signals are shown in Figure 12c at 200 kHz and Figure 12e at 430 kHz to confirm the frequency of the probe operation. The signals presented at high frequency (430 kHz in Figure 12d,e) indicate that this directional operation is observed, and the waves propagated in the expected direction. Furthermore, the directionality of the propagating wave works with both the SH0 and SH1 modes. On the other hand, the lower frequency 200-kHz signals still predominately propagated toward the near edge of the plate. Finally, additional experiments not shown here demonstrated that unidirectional transduction is feasible in a wider frequency range (150–700 kHz) with some minor adjustments of the permanent magnet position relative to the strip edge. At the present stage, the unidirectional transducers have the following advantages:

- 50% lower power consumption because only one channel pulser is needed
- Broadband (200–700 kHz) signal generation/reception without changing directionality
- Reduced sensitivity to variations in coupling when used for SHM applications, because only one sensing element with a relatively small footprint is coupled instead of two in alternative designs
- Reduced instrument complexity, because only one channel is required.

## 8. Conclusions

A review of the reversed Wiedemann effect and how it relates to sensor design was presented. It was shown that direct Wiedemann effect sensors have a bias that is perpendicular to the wave propagation direction, which creates some engineering challenges. Reversed Wiedemann sensors utilizing swapped orientations of static and dynamic magnetic fields allow strong biasing field strength with rare earth magnets and more efficient solenoidal coil designs. Some practical applications of reversed Wiedemann MsT were discussed.

Two new sensor concepts using the reversed Wiedemann effect were presented: broad frequency bandwidth probes and unidirectional probes. The broader frequency concept uses high bias and dynamic field strengths to generate multiple order odd harmonics. Unidirectional transduction capability from a single-winding sensor was demonstrated using a biasing magnet offset from the center of the iron cobalt strip. Tests conducted at a 300-kHz center frequency indicated that the effect of unidirectional operation is related to the width of the excitation area, which determines the frequency of unidirectional wave propagation. Results in the frequency range from 200 kHz (SH0 mode) to 430 kHz (SH0 and SH1 wave modes) without a repositioning of the biasing magnet were presented. These unidirectional probes can be operated by a single channel instrument, which should allow for the use of lower cost and more compact electronics.
